# At‐risk alcohol users have disrupted valence discrimination during reward anticipation

**DOI:** 10.1111/adb.13174

**Published:** 2022-04-07

**Authors:** Mica Komarnyckyj, Chris Retzler, Zhipeng Cao, Giorgio Ganis, Anna Murphy, Robert Whelan, Elsa Florence Fouragnan

**Affiliations:** ^1^ Centre for Cognition and Neuroscience University of Huddersfield Huddersfield UK; ^2^ School of Psychology Trinity College Dublin Dublin Ireland; ^3^ Department of Psychiatry University of Vermont College of Medicine Burlington Vermont USA; ^4^ School of Psychology University of Plymouth Plymouth UK; ^5^ Brain Research Imaging Centre, Faculty of Health University of Plymouth Plymouth UK

**Keywords:** alcohol, AUDIT, EEG, humans, machine learning, reward

## Abstract

Alcohol use disorder is characterised by disrupted reward learning, underpinned by dysfunctional cortico‐striatal reward pathways, although relatively little is known about the biology of reward processing in populations who engage in risky alcohol use. Cues that trigger reward anticipation can be categorized according to their learnt valence (i.e., positive vs. negative outcomes) and motivational salience (i.e., incentive vs. neutral cues). Separating EEG signals associated with these dimensions is challenging because of their inherent collinearity, but the recent application of machine learning methods to single EEG trials affords a solution. Here, the Alcohol Use Disorders Identification Test (AUDIT) was used to quantify risky alcohol use, with participants split into high alcohol (HA) (*n* = 22, mean AUDIT score: 13.82) and low alcohol (LA) (*n* = 22, mean AUDIT score: 5.77) groups. We applied machine learning multivariate single‐trial classification to the electroencephalography (EEG) data collected during reward anticipation. The LA group demonstrated significant valence discrimination in the early stages of reward anticipation within the cue‐P3 time window (400–550 ms), whereas the HA group was insensitive to valence within this time window. Notably, the LA, but not the HA group demonstrated a relationship between single‐trial variability in the early valence component and reaction times for gain and loss trials. This study evidences disrupted hypoactive valence sensitivity in the HA group, revealing potential neurophysiological markers for risky drinking behaviours which place individuals at‐risk of adverse health events.

## Graphical abstract

Here, we used a machine learning approach to disentangle two dimensions of reward anticipation—valence and salience—in young adults with and without hazardous drinking behaviours. Within the cue‐P3 time window, valence discriminator performance (i.e., Az values) was significantly higher in the low risk alcohol use (LA) group compared with the hazardous drinking (HA) group. This study evidences disrupted valence but intact salience sensitivity in the at‐risk group, revealing potential neurophysiological markers for vulnerability to AD.

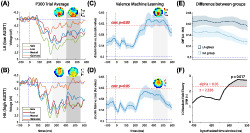

## 1 INTRODUCTION

Alcohol use disorder (AUD) is characterised by disrupted reward processing, underpinned by dysfunctional cortico‐striatal reward pathways.^1^, ^2^, ^3^ An abundance of research has identified reward circuitry alterations that regulate the development and persistence of AUD.^2^, ^3^, ^4^, ^5^ However, relatively little is known about the biology of reward processing in non‐disordered populations who exhibit risky drinking behaviours placing them at‐risk of future adverse health events.^6^ Knowing more about these at‐risk drinkers could facilitate early prevention and intervention strategies thus reducing overall alcohol related harm in society.^7^

**FIGURE 1:**
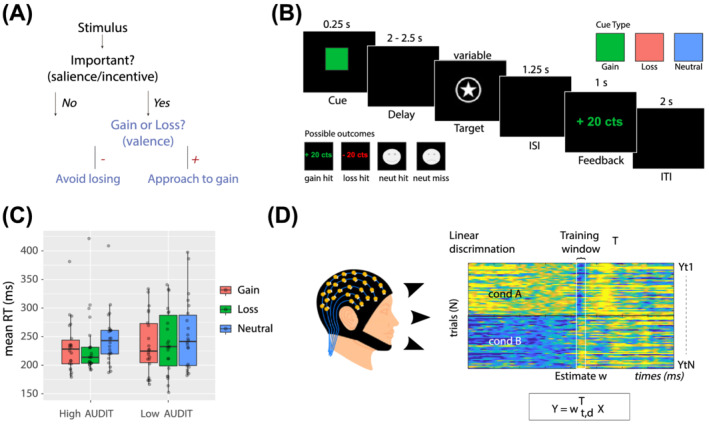
Experimental design, RT analysis and single‐trial discriminant component maps. (A) Flowchart representation of stimulus type, illustrating the difference between valence and salience. (B) Schematic representation of the experimental paradigm. On each trial, one of three cue symbols was presented for 0.25 s indicating if participants could win or lose 20cents, or if the trial would have no impact on earnings (i.e., neutral). Following a jittered delay of 2–2.5 s, a square target was presented. A staircase algorithm adapted the target duration attempting to fix accuracy at ~66% within each trial type. Following a delay of 1.25 s, feedback was shown for 1 s. A jittered inter‐trial interval of 2 s was presented before the next trial began. (C) RT results for both groups are presented together and are visualised using box and whisker plots, including the median, two hinges and two whiskers. Individual participant results are plotted as separate data points (*N* = 22 per group). *Note*: Any points which go beyond the end of the whiskers are outliers. (D) Summary of our single‐trial EEG analysis. Example of a discriminant component map resulting from our single‐trial linear discrimination analysis. The panels represent the discriminator amplitudes for the valence component for monetary loss (top) and loss cue (bottom) trials, using the training window shown by the vertical bars labelled training window

Reward processing encompasses two distinct phases. The first phase, reward anticipation, arises when a cue with learnt valence (i.e., positive vs. negative) and motivational salience (i.e., incentive vs. neutral) is encountered (Figure 1A).^8^ The second phase, reward outcome, happens when an outcome is delivered that generates a prediction error, which ultimately triggers learning.^9,10^ Reward outcomes have been extensively studied using both functional magnetic resonance imaging (fMRI) and electroencephalography (EEG). fMRI studies of AUD have generally shown increased activation in a distributed reward network, including the ventral striatum (VS), during reward outcome.^2^ Our prior EEG investigation with at‐risk drinkers concurred with fMRI findings in AUD, demonstrating an elevated neural response during the outcome phase of reward processing. Specifically, we found at‐risk drinkers had increased *feedback‐P3* amplitude (a positive‐going event related potential [ERP] component, peaking at centro‐parietal electrodes around 400 ms post feedback onset), which was related to reward prediction error.^11^ Notably, other research groups found alcohol and substance dependence to be associated with disrupted reward learning at time of outcome; however, this was characterised by reduced amplitudes for feedback‐related ERPs.^12^, ^13^

During reward anticipation, however, fMRI studies have shown hypoactivation of the main areas of the reward circuit in AUD^3^, ^4^, ^5^ although brain activity during this decision period in populations exhibiting risky drinking behaviours has not yet been investigated. The present study builds upon our prior EEG work,^11^ providing a complete picture of both phases of reward processing, by evaluating the neural dynamics of reward anticipation in at‐risk drinkers. Here, we used an EEG version of the monetary incentive delay (eMID) task.^11^, ^14^ The millisecond temporal resolution of EEG enabled full characterisation of the time course of valence and salience dimensions of reward anticipation, which have been shown to engage distinct neural networks in prior fMRI meta‐analyses.^8^, ^15^ We split participants into two groups using the Alcohol Use Disorders Identification Test (AUDIT).^16^ Participants with high AUDIT (HA) scores formed a group of at‐risk hazardous drinkers, and those with low AUDIT (LA) scores formed the control group.

Studying the valence and salience dimensions of reward in a single EEG study is challenging because of the inherent collinearity of the neural signals under investigation.^17^ These challenges may be tackled with machine learning (ML) methods applied to high density EEG data that have been successful at disentangling valence and salience at reward outcome and reliably extracting their related single‐trial EEG information.^18^ By decoding experimental conditions using whole brain activity, and therefore avoiding the use of a priori electrode selection, multivariate ML offers several analytical advantages over traditional univariate statistics, including the preservation of single trial information and an enhanced signal‐to‐noise ratio (SNR).

In conventional ERP time‐locked EEG analyses, the data are averaged over trials to increase the amplitude of the signal correlated with neural processes relative to noise, masking information contained in single trials at specific time points. The higher SNR of the ML approach facilitates more reliable extraction of single trial neural information, which can be related to variable behavioural responses that are also changing on a trial‐by‐trial basis.^19^ Based on the aforementioned advantages, the present study combines ERP analyses with a multivariate ML approach to help identify neurophysiological markers of reward anticipation in hazardous at‐risk drinkers.

eMID studies have been informative about the time course of key anticipatory events. Relevant ERPs include the *cue‐P3* and the *contingent negative variation* (CNV) slow wave.^20^ The cue‐P3 is a broad positive wave, peaking around 350–550 ms post stimulus that is related to novelty, attention and arousal.^21^, ^22^ Moreover, it is thought to be generated by the central dopaminergic system.^23^ Importantly, the cue‐P3 is a potential neural marker for reward disruption in AUD since its amplitude correlates with ventral striatum (VS) signal during reward processing.^24^ Prior eMID literature with healthy participants consistently demonstrated cue‐P3 valence sensitivity (i.e., enhanced neural processing for the positively valenced gain cues compared with the negatively valenced loss cues).^24^, ^25^, ^26^, ^27^ We therefore hypothesised that the LA group would have larger cue‐P3 amplitudes for gain compared with loss cues and significant gain versus loss ML discrimination, termed *A*_*z*_ *value*, within this time window. Considering fMRI MID studies found diminished valenced sensitivity in AUD^5^ and hazardous at‐risk drinkers (>16 days of drinking per month),^28^ we hypothesised the HA group compared with the LA group would exhibit reduced cue‐P3 valence sensitivity and reduced gain versus loss A_z_ value within this time window.

The CNV is a slow negative wave occurring in time between a stimulus which prompts preparatory processes, and another event which requires a motor response.^29^ It is thought to reflect motivation associated with intention to perform an act.^30^ Previous research suggests that the CNV originates in the anterior cingulate cortex (ACC), supplementary motor area (SMA) and bilateral thalamus, and is commonly associated with alertness and salience.^10^, ^31^, ^32^ Some eMID studies with healthy participants report the CNV to be insensitive to experimental manipulation.^24^, ^26^, ^27^, ^33^ Others report salience sensitivity (i.e., enhanced neural processing for the high salience incentive cues compared with the low salience neutral cues) with task modulation occurring in the late stages of anticipatory processing, during preparation for the motor response to the eMID target.^22^, ^25^

Importantly, the CNV is relevant for at‐risk drinkers: healthy young adults who first consumed alcohol during puberty have enhanced CNV amplitudes compared with those who begin consumption post puberty.^34^ We therefore predicted that in the late CNV window, 200 ms before target onset,^22^, ^25^ the HA group would demonstrate enhanced salience sensitivity compared with the LA group reflecting altered motivational mechanisms. This would be evidenced by a greater difference in neural processing between incentive and neutral cues.

A full list of testable hypotheses and further exploratory analysis for the P2 and N2 components are detailed in Supplementary Information (Table S1).

## 2 METHODS

### 2.1 Participants

The final sample consisted of 44 participants (20 female), with a mean age of 23.77 years (range 18–32 years) (see Table 1 for group comparison of demographic data). Participants were recruited at university campuses via posters as part of a wider study on substance use. Participants were subsequently telephone screened to determine eligibility. Exclusion criteria included being less than 18 years old; any physical disability or learning difficulties that would impact task performance (e.g., motor impairment); any history of major axis I mental‐health illness (excluding depression), as defined by the Diagnostic and Statistical Manual of Mental Disorders (DSM); any history of head trauma (including concussion) or stroke; and regular cannabis or drug use (more than twice a month). Eligible participants were required to complete online questionnaires (including AUDIT and the Drug Abuse Screening Test [DAST]^35^). Based on AUDIT scores, we invited a subset of participants to attend the 2‐h EEG laboratory session (see Table 1), with the goal of recruiting equal numbers of participants with high and low AUDIT scores. Participants were compensated with €20 for EEG session completion, and with up to €10 for travel expenses. Alternatively, eligible participants could opt for compensation with course credit. All participants gave written informed consent to participate. The study was approved by the Trinity College Dublin School of Psychology Ethics Committee and the University College Dublin School of Psychology Ethics Committee.

**TABLE 1:** Demographic data and clinical measures

	HA	LA	*p*
Sample size	22	22	
AUDIT total, mean (SD)	13.82 (3.57)	5.77 (2.18)	<0.001
Gender, male/female	11/11	13/9	0.367
Age, y, mean (SD)	23.91 (4.41)	23.64 (4.67)	0.635
Education, y, mean (SD)	16.73 (2.21)	15.59 (2.99)	0.225
Smoker/non‐smoker, *n*	10/12	12/10	0.364
DAST drug use total, mean (SD)	2.64 (3.57)	4.55 (7.13)	0.971

*Note*: Group differences for continuous variables were carried out with independent sample *t* tests; however, gender and smoking status were tested using chi‐squared tests.

Abbreviations: AUDIT, Alcohol Use Disorder Identification Test; DAST, Drug Abuse Screening Test; HA, participants with high alcohol use based on AUDIT score; LA, participants with low alcohol use based on AUDIT score; RT, RTs in milliseconds; SD, standard deviation; y, years.

### 2.2 Alcohol and substance‐use measures

The AUDIT was used to assess the nature and severity of any alcohol misuse, and to quantify risk on a scale from low‐level to hazardous drinking. The 10 AUDIT questions are each scored from 0 to 4, giving a maximum total score of 40. Based on diagnostic guidelines for AUDIT, participants with a total AUDIT score of greater than 8 were classified as having hazardous drinking behaviours and formed a high AUDIT (HA) group who have a higher risk of developing AUD. Following the method in our previously published study, participants with a total AUDIT score of 8 or below were classified as low risk and formed a low AUDIT (LA) control group.^11^ Twenty‐two participants were recruited in each group. Psychoactive drug use was assessed using the DAST. Smokers were defined as people who reported smoking at least one cigarette per day in the 30 days prior to testing.

### 2.3 Experimental design

Participants performed an eMID task (Figure 1B), adapted from that originally employed by Knutson for use with fMRI.^14^ During the anticipation phase of the task, participants were presented with a cue symbol for 250 ms, which informed them of the type of trial they were about to perform. A green square indicated the potential to win 20 cents (i.e., gain condition); a red square indicated the potential to lose 20 cents (i.e., loss condition); and a blue square indicated there would be no financial outcome (i.e., neutral condition). This was followed by a target anticipation period where a blank screen was displayed for 2000 to 2500 ms (see Figure 1 for the full experimental design).

In the following target phase, a white solid pentagram in a circle was displayed for a variable duration (the response interval was varied based on task performance; potential range = 100–1250 ms). Before commencing the task, participants were asked to respond to the target as quickly as possible with the index finger of their dominant hand via a wired controller. For the incentive cues, hitting the target (which was presented for a variable duration, the “response hit interval”) resulted in monetary gain, or avoidance of monetary loss. An adaptive algorithm, based on target response hit interval, was employed with the goal of providing 66% positive feedback across all conditions. The response interval for the target was reduced if the success rate exceeded 66% (increasing task difficulty) and lengthened if the success rate was below 66% (reducing task difficulty). Participants were not made aware of the adaptive algorithm as this could undermine their motivation to perform well in the task. The aim of the algorithm is to keep the subjective difficulty of the task consistent across participants, despite variation in response time. The adaptive algorithm functioned as intended, with participants achieving a mean success rate of 66.3% (std. = 1.94) and no participants were excluded due to low success: fail ratio. Following offset of the target stimulus, a blank screen was presented for 1200 ms.

In the subsequent outcome phase of the task feedback was displayed for 1000 ms. For gain cues, the feedback shown was “+20 ct” when the target was hit, and a sad cartoon face when the target was missed. For loss cues, the feedback shown was “−20 ct” when the target was missed and a smiley cartoon face when the target was hit. For the neutral cues, the feedback was a smiley or sad cartoon face when the target was hit or missed, respectively. Each trial was separated by a 2000‐ms inter‐trial interval. Analyses from the outcome phase were reported previously.^11^ Each participant completed 48 trials per condition (gain, loss, neutral; 144 trials total). Participants completed a practice set of 30 trials prior to the full task.

### 2.4 EEG data acquisition and pre‐processing

An ActiveTwo Biosemi system was used to record EEG data (1024‐Hz sampling rate), with 64 scalp electrodes positioned according to the 10–10 system. Participants sat in a darkened, sound attenuated room, 1.05 m in front of a Dell cathode ray tube computer monitor (75‐Hz refresh rate; 1024 × 768 pixels), resulting in each centimetre subtending 0.55 degrees of visual angle. Horizontal and vertical electro‐oculograms associated with eye movement were recorded bilaterally using four additional electrodes. These were placed approximately 2 cm below the eyes and from the outer canthi. Two additional electrodes were located on the mastoids.

EEG data were pre‐processed using the EEGLAB toolbox^36^ and the Fully Automated Statistical Thresholding for EEG Artifact Rejection (FASTER) plug‐in,^37^ as in Cao et al.^11^ The raw EEG data were band pass filtered from 0.1 to 95 Hz, and a 50‐Hz notch filter was applied. The data were then average‐referenced across all scalp electrodes. Epoching was performed from 500 ms pre‐stimulus to 2000 ms post‐stimulus, locked to the cue, target, and feedback onset. Artifact detection is described in Supplementary Information. Subsequently, data were low‐pass filtered at 30 Hz.

### 2.5 Event related potential analyses

The cue‐P3a and cue‐P3b were quantified over a parietal group of electrodes (P1, P2, POz, Pz)^25^ and measured as the mean amplitude between 250–400 ms and 400–550 ms after cue onset, respectively. The P2 and N2 were quantified over a frontal group of electrodes (FC1, FC2, FCz, Fz)^22^ and measured as the mean amplitude between 160–210 ms and 210–310 ms after cue onset, respectively. The CNV was measured as the mean amplitude 200 ms prior to target presentation (1800 to 2000 ms post cue onset) over a central group of electrodes (C1, C2, Cz, FCz).^25^ ERPs were calculated using all remaining epochs following artifact rejection (see Supplementary Information).

All ERP statistical analyses were carried out using R.^38^ We first conducted one‐way repeated measures ANOVAs on mean ERP amplitudes, with condition (gain, loss, and neutral) as the within‐subjects factor, for the LA group and HA group individually. These tests were conducted for all ERP components separately (including cue‐P3a, cue‐P3b, P2, N2, CNV). This enabled us to validate the task by allowing comparison with prior EEG MID literature which has focused on reward anticipation in healthy subjects. To assess group differences, mixed ANOVA tests were then conducted on mean ERP amplitudes with condition as the within‐subjects factor and group as the between‐subjects factor. Again, separate tests were used for all ERP components. ERP data were checked using Mauchley test for sphericity and Greenhouse–Geisser correction was applied when any violation occurred. Significant ANOVA tests were investigated with Tukey's honest significant difference post‐hoc test to confirm which conditions were significantly different from each other. The significance level was set at *p* < 0.05 for all statistical tests completed.

Based on the a priori hypotheses of valence sensitivity (i.e., gain amplitudes > loss amplitudes) we also evaluated whether gain‐minus‐loss ERP difference waves were sensitive to between group differences (see Supplementary Information). This alternative analysis strategy can be more effective at isolating components of interest, by eliminating common operations between two conditions.^39^

### 2.6 Machine learning analyses

We used single‐trial, multivariate discriminant analysis on our epoched EEG data^10^, ^18^, ^40^ to perform binary discriminations along the dimensions of valence and salience during the anticipatory phase of the MID task (Figure for an illustration of the analysis). For each participant, we estimated linear weightings of the EEG electrode signals (i.e., spatial weighting vectors) across the whole brain that provided maximal discrimination between (1) gain versus loss cue trials (valence) and (2) incentive (gain and loss) versus neutral cue trials (salience) (Equation 1). For the salience discrimination, to avoid any confound due to velocity in motor preparation, we extracted incentive trials that were matched to neutral trials based on the distribution of reaction times (RTs). For this, we used the distribution of RTs in the neutral trials approximated with a Gaussian distribution and extracted trials from the gain and loss conditions to match this distribution. We ensured there were an equal number of incentive compared with neutral trials and that the mean and variance of these two incentive and neutral distributions were in the same range (mean = 40.54 trials, std. = 3.99).

The multivariate discriminant analysis was conducted over several temporally distinct training windows to identify temporally distinct neural components associated with valence and salience (Figure 1D). The training windows had a fixed window length of δ = 60 ms and onset times τ varying from −200 to 2000 ms relative to cue onset (increasing in 10‐ms increments). We used a regularized Fisher discriminant to estimate the spatial weighting vectors $w\left(\tau \right),$ which maximally discriminates between EEG electrode signals $x\left(t\right)$, for two groups of interest (i.e., gain vs. loss trials for the valence dimension, and incentive versus neutral trials for the salience dimension):

(1) $$y_{i}\left(\tau \right)=\frac{1}{N}\sum_{t=\tau -N/2}^{t=\tau +N/2}w{\left(\tau \right)}^{T}\;x_{i}\left(t\right)\;$$

The pre‐processed EEG data $x\left(t\right)$ are a multidimensional *D* × *T* matrix, with *D* = electrodes, *T* = time samples, and with *N* = total number of trials. The resulting one‐dimensional “discriminating component” $\;y_{i}\left(\tau \right)$ is produced by applying the spatial weighting vectors $w\left(\tau \right)\;$ to the single trial EEG data $x_{i}\left(t\right)$, and linearly integrating single‐trial information across spatially distributed electrodes. It can therefore be conceptualised as an individual whole brain channel that reflects single trial variability (STV) in neuronal response associated within each group of interest. Notably, any shared activity between conditions is removed. The “discriminating component” $\;y_{i}\left(\tau \right)$ has enhanced SNR compared with individual channel data, due to less interference from physiological and environmental noise which do not contribute to the binary discrimination.^40^

The spatial weighting vectors $w\left(\tau \right)\;$ for each onset time τ were calculated as follows: $w=S_{c}\left(m_{2}-m_{1}\right)\;$ with $m_{i}\;$ being the mean for each group i and $S_{c}=\frac{1}{2}\left(S_{1}-S_{2}\right)\;$ is the common covariance matrix—the average of the covariance matrices for each group, $\;S_{i}=1\left(N-1\right)\sum_{j=1}^{N}\left(x_{j}-m_{i}\right){\left(x_{j}-m_{i}\right)}^{T}\;$. Additionally, we regularized the covariance matrices for each group in order to avoid any estimation errors, following the equation: $\;{\tilde{S}}_{i}=\left(1-\lambda \;\right)\;S_{i}+\lambda vI$, with $\lambda \in \left[0,1\right]\;$ being the regularization term and v the average eigenvalue of the original $\;S_{i}$ (i.e., trace ( $S_{i})/D$). For λ=0, no regularization is being applied whereas λ=1 assumes spherical covariance matrices. For each participant, we optimized λ at each onset time during the entire period following the cue presentation based on discriminator performance (see below) using grid search in increments of 0.01.

Performance of the discriminator was quantified at each time window of interest by calculating the area under a receiver operating characteristic (ROC) curve (termed *A*_*z*_ value) using a leave‐one‐out trial (LOO) cross validation approach. Significance of the discriminator performance was assessed by permutation testing using the leave‐one‐out trial procedure after randomizing the labels associated with each trial. We repeated this randomisation procedure 1000 times to produce a probability distribution for A_z_ and estimate the A_z_ value leading to a significance level of *p* < 0.05. Due to the linearity of the model, we also computed scalp topographies of the significant discriminating components output from Equation (1 estimating forward models using Equation (2, which describes the electrical coupling of the single‐trial component amplitudes $y\left(\tau \right)\;$ and the observed data x.

(2) $$a_{\tau }=\frac{x\left(\tau \right)\;y\left(\tau \right)\;}{y{\left(\tau \right)}^{T}y\left(\tau \right)}$$

### 2.7 Between group comparison of machine learning results

SPM1d version 0.4.7 (MATLAB open‐source software, available at http://www.spm1d.org/) was used to statistically compare group averaged A_z_ values (i.e., ML discriminator performance). SPM1d is similar to the conventional SPM (statistical parametric mapping) approach to inferential statistics but is designed for one‐dimensional data. This toolbox provides the following advantages: (1) it improves on Bonferroni correction for multiple comparisons by using random field theory to account for covariance between scalar components at each timepoint; (2) the critical threshold (test statistic) accounts for timeseries smoothness and length; (3) cluster specific *p* values are calculated, which indicate the probability of producing supra‐threshold clusters (i.e., multiple adjacent timepoints exceeding the critical threshold [α] of 0.05).^41^, ^42^, ^43^ Calculation of SPM{t} is detailed in Supplementary Information.

Specifically, in accordance with our predictions, we conducted one‐tailed unpaired *t* tests which evaluated if (1) the A_z_ values for valence discrimination (i.e., ability to differentiate between gain and loss trials) were greater in the LA group compared with the HA group, from 400–550 ms after cue onset; and (2) the A_z_ values for salience discrimination (i.e., ability to differentiate between incentive and neutral trials) were greater in the HA group compared with the LA group, from 1800 to 2000 ms after cue onset.

### 2.8 Relationship between behaviour and single trial variability

We extracted the single‐trial variability (STV) for each individual participant, at the group averaged time of maximum significant discrimination (t) for the valence (STV.Valence(t)) and salience (STV.Salience(t)), thus identifying the point with most trial‐by‐trial variance in the data. Our aim was to evaluate the relationship between behaviour and brain activation during the decision‐making process, by relating STV to the trial‐to‐trial variability in RTs with a multiple regression model. To do so, we first extracted the discriminator output with dimensions *T* × *N*, where *T* is the number of EEG samples and *N* the number of trials. Second, to extract robust STV, yτ, we averaged all samples within our time window, to obtain:

(3) $${\bar{y}}_{\tau -i}=\frac{1}{T}\;\sum_{j=1}^{T}y_{\tau -\mathrm{ij}}$$

where *i* is the trials index and *j* the EEG samples index. Once we had extracted our STV per participant, *y*_*τ*_, we used the STV as parametric regressors to see if they predicted variability in upcoming RTs.

In order to control for potential confounding factors in the regression, specifically trial order and outcome, we also computed the trial order (*trial_order*) and the outcome of the task (correct [+1]/incorrect [−1], *trial_outcome*) and added these as regressors of non‐interest in our regression analysis.

For valence, we used the following equation to predict RTs based on the single trial discriminator amplitudes of gain versus loss cues, in a mixed‐effect model such as

(4) $$\mathrm{RTs}\;\sim \;1+\;\beta *\mathrm{STV}.\;\text{Valence}\left(t\right)+\beta *\text{trial}\_\text{order}+\beta *\text{trial}\_\text{outcome}$$

For salience, we used the following equation to predict RT based on the discriminator amplitudes of incentive versus neutral cues, in a mixed‐effect model such as:

(5) $$\mathrm{RTs}\;\sim \;1+\beta *\mathrm{STV}.\;\text{Salience}\left(t\right)+\beta *\text{trial}\_\text{order}+\beta *\text{trial}\_\text{outcome}$$

To establish a significant trial‐by‐trial association between RTs and discriminator output we tested whether the regression coefficients resulting from all participants came from a distribution with mean greater than zero (using a one‐tailed *t* test).

## 3 RESULTS

### 3.1 Behavioural data

The 3 (condition) × 2 (group) mixed ANOVA revealed a main effect of condition on RT, *F*(1.94, 81.36) = 14.16, *p* < 0.01. Tukey post hoc tests showed significantly longer RTs for the neutral compared with the gain (*p* < 0.0001) and loss (*p* = 0.0003) conditions (Figure 1B). There were no significant group effects or interactions.

### 3.2 ERP results

For gain‐minus‐loss difference wave and exploratory component (P2 and N2) results see Supplementary Information.

#### 3.2.1 Cue‐P3a

**FIGURE 2:**
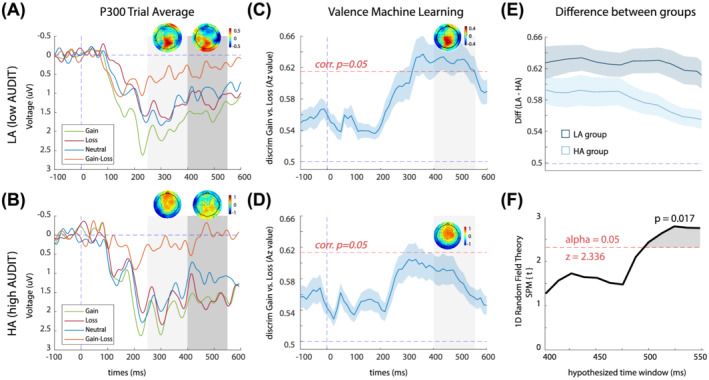
Cue‐locked valence ERPs and discrimination results. (A) Average ERP components for the LA group, computed over parietal electrodes [P1, P2, POz, Pz]. Red, blue, green and orange traces represent loss, neutral, gain and gain–loss trials, respectively. The first and second grey‐shaded bars depict the time windows for the Cue‐P3a and Cue‐P3b, respectively. Scalp topographies are included for peaks of the gain‐minus‐loss difference waves within these time windows (265–305 ms and 405–465 ms). Note that ERPs are plotted with the negative *y* axis pointing up. (B) Average ERP components for the HA group, computed over parietal electrodes [P1, P2, POz, Pz]. Red, blue, green and orange traces represent loss, neutral, gain and gain–loss trials, respectively. The first and second grey‐shaded bars depict the time windows for the Cue‐P3a and Cue‐P3b, respectively. Scalp topographies are extracted for the peaks of the gain‐minus‐loss difference waves within these time windows (265–305 ms and 405–465 ms). (C) Single‐trial discriminator performance (Az) between gain and losses as a function of cue‐locked time for the LA group. Results are averaged over all participants (mean line in blue ±se across participants, represented by the shaded blue area). The dotted red line represents the Az leading to a significance level of *p* = 0.05. The grey boxes represent the time windows of interest used for the STV analysis. The forward model is presented at 435 ms (scalp maps, A). (D) Single‐trial discriminator performance (Az) between gain and losses as a function of cue‐locked time for the HA group. There were no time windows reaching significance in the entire period after cue presentation. The forward model is presented at 435 ms (scalp maps, A). (E) Comparison of mean HA‐Az and LA‐Az across the window of interest (400–550 ms). (F) Results of SPM1d analysis, illustrating the magnitude of LA‐Az to HA‐Az differences (i.e., SPM{t}) across time window of interest (400–550). Grey‐shaded region indicates where there is a significant difference between HA‐Az and LA‐Az values, that is, where the critical threshold (2.336) has been crossed by the SPM{t}

For the LA group, we conducted a one‐way repeated measures ANOVA on mean cue‐P3a and found a significant main effect of condition, *F*(2, 42) = 6.69, *p* = 0.003, η_p_^2^ = 0.242 (Figure 2A). Tukey post hoc tests showed significantly larger cue‐P3a amplitudes for the gain condition compared with the loss condition (*p* = 0.014) and the gain condition compared with the neutral condition (*p* = 0.021). For the HA group, we conducted a one‐way repeated measures ANOVA on mean cue‐P3a, but the main effect of the condition did not reach significance, *F*(2, 42) = 1.45, *p* = 0.247, η_p_^2^ = 0.064 (Figure 2B).

The 3 (condition) × 2 (group) mixed ANOVA revealed no significant differences between the groups for the cue‐P3a amplitude, *F*(1, 42) = 0.28, *p* = 0.600, η_p_^2^ = 0.007. There was, however, a significant main effect of condition on cue‐P3a amplitude, *F*(2, 84) = 5.81, *p* = 0.004, η_p_^2^ = 0.122. Tukey post hoc tests showed significantly larger cue‐P3a amplitudes for the gain condition compared with the loss condition (*p* = 0.010) and the gain condition compared with the neutral condition (*p* = 0.019).

#### 3.2.2 Cue‐P3b

For the LA group, we conducted a one‐way repeated measures ANOVA on mean cue‐P3b and found a significant main effect of condition, *F*(2, 42) = 4.23, *p* = 0.021, η_p_^2^ = 0.168 (Figure 2A). Tukey post hoc tests showed significantly larger cue‐P3b for the gain condition compared with the neutral condition (*p* = 0.003). For the HA group, we conducted a one‐way repeated measures ANOVA on mean cue‐P3b, but the main effect of the condition was not significant, *F*(2, 42) = 1.92, *p* = 0.065, η_p_^2^ = 0.122 (Figure 2B).

The 3 (condition) × 2 (group) mixed ANOVA revealed no significant differences between the groups for the cue‐P3b amplitude, *F*(1, 42) = 0.38, *p* = 0.541, η_p_^2^ = 0.009. There was, however, a significant main effect of condition on cue‐P3b amplitude, *F*(2, 84) = 5.91, *p* = 0.004, η_p_^2^ = 0.123. Tukey post hoc tests showed significantly larger cue‐P3b amplitudes for the gain condition compared with the neutral condition (*p* = 0.003).

#### 3.2.3 CNV

For the LA group, we conducted a one‐way repeated measures ANOVA on mean CNV, but the main effect of the condition did not reach significance, *F*(2, 42) = 1.10, *p* = 0.343, η_p_^2^ = 0.050 (Figure 4A). For the HA group, we conducted a one‐way repeated measures ANOVA on mean CNV and found a significant main effect of condition, *F*(2, 42) = 5.73, *p* = 0.006, η_p_^2^ = 0.214 (Figure 4B). Tukey post hoc tests showed significantly larger CNV for the loss condition compared with the neutral condition (*p* = 0.005).

The 3 (condition) × 2 (group) mixed ANOVA revealed no significant difference between the groups for the CNV amplitude, *F*(1, 42)= 0.54, *p* = 0.467, η_p_^2^ = 0.013. There was a significant main effect of condition on CNV amplitude, *F*(2, 84) = 6.04, *p* = 0.004, η_p_^2^ = 0.126. Tukey post hoc tests showed significantly larger CNV amplitudes for the gain condition compared with the neutral condition (*p* = 0.023) and loss condition compared with the neural condition (*p* = 0.016).

### 3.3 Machine learning results

#### 3.3.1 Valence

For the LA group, we observed a wide temporal window of significant A_z_ value for valence discrimination: between 333 and 512 ms after cue onset (Figure 2C). The forward model (scalp map) extracted from the midpoint (423 ms) of the significant valence component was concentrated over parietal electrodes, aligned with reported neural generators of the cue‐P3 ERP component.^21^ For the HA group, the mean A_z_ value for valence discrimination did not reach significance at any time point between cue onset and target presentation (Figure 2D). A_z_ values were significantly larger in the LA group compared with the HA group (Figure 2E,F), from 480 to 550 ms (Figure 2F; *p*_cluster_ = 0.017).

#### 3.3.2 Trial‐by‐trial RT indexed by valence component

**FIGURE 3:**
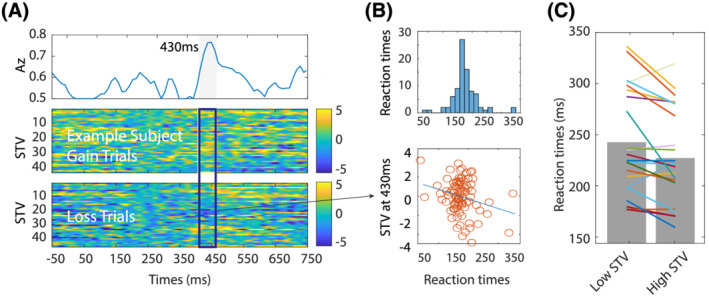
STV in the valence component predicts RT sin the LA group. (A) Single‐trial discriminator performance (Az) between gain and losses as a function of cue‐locked time for a representative participant in the LA group (top panel). Single‐trial PE discriminant component maps for gain and loss trial for the same an exemplar participant (middle and bottom panels respectively). (B) Representation of the RT distribution and scatter plot between the RTs and the STV for the exemplar participant. (C) EEG STV component amplitudes separated by slow and fast RTs (the bins were created by splitting the RTs in equal groups)

The STV in our valence component was predictive of RT triggered by the cue (Figure 3A,B depicts an individual example of Valence STV, RT distributions and relationship between the two). Our regression analysis included STV for each participant, extracted at the group average peak of maximum discrimination, within our hypothesised time window for valence processing (400–550 ms) (see Figure 3C). Specifically, there was a significant negative relationship between RTs and the STV in the valence component in the LA group; the estimated regression coefficients (See Method's β1's in Equation 4) were significantly different from zero *t*(21) = −2.14, *p* = 0.044. In other words, the larger the differentiation in neural processing between gain and loss cues, the faster the response times to the target. Furthermore, for our regressors of non‐interest, we found no effect of trial order on RTs, *t*(21) = 0.28, *p* = 0.78, and a strong effect of outcome on RTs, *t*(21) = −5.8, *p* = 7.6e−06, that did not impact the nature of the relation between neural data and RTs. This analysis was also conducted in the HA group but failed to reach significance. An unpaired *t* test comparing the beta coefficients output from the regression analyses conducted within each group did not reach significance *t*(86) = −0.828, *p* = 0.410.

#### 3.3.3 Salience

**FIGURE 4:**
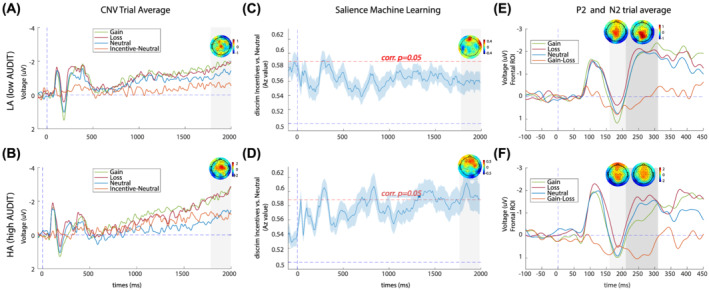
Cue‐locked salience ERPs and discrimination results. (A) Average ERP components for the LA group, computed over central electrodes [C1, C2, Cz, FCz]. Red, blue, green and orange traces represent loss, neutral, gain and incentive‐neutral trials respectively. The grey shaded bar depicts the time window for the late CNV. A scalp topography has been included for the peak of the incentive‐minus‐neutral difference wave within this time window (1800–1850 ms). Note that ERPs are plotted with the negative *y* axis pointing up. (B) Average ERP components for the HA group, computed over central electrodes [C1, C2, Cz, FCz]. Red, blue, green and orange traces represent loss, neutral, gain and incentive‐neutral trials respectively. The grey shaded bar depicts the time window for the late CNV. A scalp topography has been included for the peak of the incentive–minus‐neutral difference wave within this time window (1800–1850 ms). (C) Single‐trial discriminator performance (Az) between incentive and neutral trials as a function of cue‐locked time for the LA group. Results are averaged over all participants (mean line in blue ±se across participants, represented by the shaded blue area). The dotted red line represents the Az leading to a significance level of *p* = 0.05. There were no time windows reaching significance in the late period after cue presentation. The forward model is presented at 1884 ms (scalp maps, A). (D) Single‐trial discriminator performance (Az) between incentive and neutral trials as a function of cue‐locked time for the HA group. The grey boxes represent the time windows where the discrimination reaches significance. The forward model is presented at 1884 ms (scalp maps, A). (E) Average ERP components for the LA group, computed over frontal electrodes [FC1, FC2, FCz, Fz]. Red, blue, green and orange traces represent loss, neutral, gain and gain–loss trials, respectively. The first and second grey‐shaded bars depict the time windows for the P2 and N2, respectively. Scalp topographies are included for peaks of the gain‐minus‐loss difference waves within these time windows (175–195 ms and 230–260 ms). (F) Average ERP components for the HA group, computed over frontal electrodes [FC1, FC2, FCz, Fz]. Red, blue, green and orange traces represent loss, neutral, gain and gain–loss trials, respectively. The first and second grey shaded bars depict the time windows for the P2 and N2, respectively. Scalp topographies are included for peaks of the gain‐minus‐loss difference waves within these time windows (175–195 ms and 230–260 ms)

For the LA group, we observed a significant early peak of salience discrimination 282 ms after cue onset (Figure 4C). The mean A_z_ LOO value for the LA group salience discrimination did not reach significance at any other time point between cue onset and target presentation. For the HA group, we observed a significant early peak of salience discrimination 294 ms after cue onset (Figure 4D). Note that there is no difference in time between the early salience components across the two groups, *t*(42) = 0.65, *p* = 0.51; thus, it is assumed to be a similar neural generator.

The HA group but not the LA group, had significant salience discrimination at later stages of reward anticipation within the time window of the CNV slow wave. The HA group exhibited a maximum significant peak of discrimination at 1846 ms. The forward models (scalp maps) extracted at this time point had a fronto‐central spatial distribution, aligned with typical generators of the early CNV slow wave.^29^ There were no group differences in salience discrimination (all clusters with an SPM{t} value less than critical threshold).

#### 3.3.4 Trial‐by‐trial RT indexed by salience component

There were no significant results from the single‐trial mixed effect regression analysis between the salience STV and RT.

## 4 DISCUSSION

### 4.1 Summary of the main findings

Here, we disentangled two dimensions of reward anticipation—valence and salience—in young adults with and without hazardous drinking behaviours. Within the cue‐P3 time window, valence discriminator performance (i.e., A_z_ values) was significantly higher in the low risk alcohol use (LA) group compared with the hazardous drinking (HA) group, indicating disrupted valence discrimination in the HA group. Additionally, we discovered a negative relationship between valence discrimination STV and RT in the LA group only, such that greater variability in the neural response between gain and loss cues led to faster response times to the target. Although there were no significant between‐group differences in our ERP and ML salience discrimination analyses, our data show a trend towards enhanced salience sensitivity in the HA group. Our findings demonstrate young adults with hazardous levels of drinking have lower neural sensitivity to valence, but not salience, during reward anticipation.

### 4.2 Hazardous drinking is associated with disrupted valence processing

The single trial machine‐learning approach revealed valence discrimination differences within the cue‐P3 time window. For the LA group, there was a sustained period of significant discrimination between gain and loss cues between 333 and 512 ms after cue onset. The spatial distribution of the single trial valence component aligned with cue‐P3 ERP scalp topographies of gain and loss anticipation in prior eMID studies.^24^, ^25^ In contrast, the HA group did not exhibit significant valence discrimination at any time point between the cue and target response. Our between group comparison demonstrated that A_z_ values were significantly larger in the LA group compared with the HA group between 480–550 ms. Valence processing, as a direct contrast between gain and loss cues, is often overlooked in MID addiction studies.^44^ However, one fMRI MID study found a lack of valence sensitivity during reward anticipation within the VS of detoxified AUD participants.^5^ Another study with non‐disordered participants also found at‐risk behaviours (>16 days of drinking per month) were associated with diminished valence sensitivity in the thalamus.^28^

Considering the ERP waveforms (see Figure 2A,B), the lack of valence discrimination in the HA may be driven by a trend towards a hyperactive response to loss cues compared with the LA group. Notably, this study found no evidence of a hypoactive response to gain cues as demonstrated in previous fMRI MID studies with AUD participants.^3^, ^4^, ^5^ A hyperactive negative valence system has also been described in a recent study comparing binge drinkers with control participants, with brain activation to loss events abnormally elevated within the hippocampus.^45^ Our study provides further support for the growing body of research demonstrating disrupted valence processing in populations with at‐risk drinking behaviours.

### 4.3 Trends towards enhanced salience sensitivity in hazardous drinkers

In the later stage of reward anticipation within the CNV time window—200 ms before target response—the LA group lacked salience sensitivity (Figure 4A,C) with no differences in neural processing between incentive and neutral cues. It remains unclear whether the CNV is reliably modulated by the eMID paradigm in healthy participants, since a number of prior studies also reported CNV insensitivity,^24^, ^26^, ^27^, ^33^ but others demonstrate modulation by salience.^22^, ^25^

There was a trend towards enhanced salience sensitivity in the HA group compared with the LA group (Figure 4A–D), but this did not reach statistical significance. The mixed model ANOVA showed increased CNV amplitudes for both gain and loss cues compared with the neutral cue. Taking into consideration the ERP waveforms (see Figure 4A,B), this effect appears to be driven by an increased response to incentive compared with neutral cues in the HA group. Furthermore, we found multiple time points of significant ML salience discrimination in the HA group (790–870 ms, 1310–1510 ms, 1538–1602, and 1782–1974 ms) (Figure 4D) which were lacking in the LA group (Figure 4C). A prior eMID study also demonstrated an elevated CNV salience response (2000–3000 ms after cue onset) in young adults deemed at‐risk for AUD based on first alcohol intake during puberty.^34^

These results point towards a possible altered hyperactive motivational mechanism in at‐risk hazardous drinkers, across the entire CNV time window, encompassing arousal, response orientation and motor preparation.^31^ The CNV is therefore highlighted as a neurophysiological marker of interest in future study of AUDs.

### 4.4 Reaction time data

Our behavioural results were consistent with prior eMID literature, with faster responses for incentive compared with neutral trials, and no difference between gain and loss trials.^22^, ^25^, ^46^ Thus, it appears that participants were motivated for the incentive conditions relative to neutral trials and that gain and loss avoidance were equally motivating. Reaction times per condition did not differ between groups despite blunted valence discrimination in the HA group. Prior monetary reward research comparing participants with AUD to healthy participants also found no differences in RT between groups, despite finding blunted VS activation during reward anticipation^4^, ^5^ and blunted feedback‐P3 during reward outcome.^5^ In contrast to the analysis of average RT, we hypothesized that the maximum valence component carried task‐relevant information, which we could exploit to predict upcoming RTs. Supporting our hypothesis, there was a significant negative relationship between RTs and the single trial variability (STV) in the valence component in the LA group only. In other words, the higher the valence of the neural response (as indexed by the EEG), the faster the LA participants responded to the target. Ultimately these results offer a link between behaviour and neuronal variability in our valence component in the LA group. We propose that trial‐by‐trial variability in RT is linked to variations in trial‐by‐trial incentive motivation, triggered by the gain and loss cues. Since we found a strong effect of trial outcome in our regression, we speculate RT variability may also by related to neuronal variability due to fluctuations in global reward state (i.e., accumulated reward over time) which indirectly effects motivation.^47^ Our regression analyses demonstrated RT could not be explained by trial order, thus variations in RT did not reflect participant fatigue as the task progressed.

### 4.5 Strengths and limitations

A strength of this study was the application of ML analyses to the data – a multivariate approach that makes use of information across the whole brain of electrodes rather than focusing on a single electrode (or small cluster of electrodes). The approach linearly combines information from all electrodes into a single channel (i.e., single trial component). By integrating across space, rather than averaging across time, SNR is increased and interference from other sources reduced, when compared with the univariate ERP method.^19^

Although there was no significant group difference in the cue‐P3 ERP valence sensitivity (i.e., gain vs. loss contrast using a trial averaged approach calculated over P1, P2, POz, Pz) (Figure 2A,B), the comparison of ML valence discrimination performance over the same time window demonstrated a significant between‐group difference. Discrepancies between the results could be a consequence of the enhanced SNR offered by the ML approach. That is, the ML analysis linearly integrated information from all trials and electrodes to determine time points of maximum discrimination between conditions (i.e., single trial valence component), whereas our ERP cue‐P3 analyses collapsed information across trials, ignoring inter‐trial variability, and was localised over a group of four parietal electrodes. The sample size was relatively modest (total *n* = 44).

### 4.6 Future directions

Chronic substance abuse overstimulates the mesolimbic pathway causing neuroadaptations which raise the natural reward threshold, contributing to the maintenance of drug‐seeking and taking behaviour.^48^ Considering severe neuroadaptation from alcohol consumption is not present in young adult nonchronic users,^49^, ^50^ it is theorised that the disrupted valence processing uncovered in this study is pre‐existing in our HA group and could be a neurophysiological marker for the development of more severe AUDs. To confirm this hypothesis further longitudinal research is required, which evaluates sensitivity to valence and salience from youth to adulthood, and its relationship with transition to AUD. Future studies could also seek to extend the analysis to other groups (e.g., to detect differences across mild, to moderate and severe AUD).

## 5 CONCLUSION

In the present study, young adults with higher AUDIT scores exhibited disrupted valence processing, but intact salience processing, compared with those with low AUDIT scores. More specifically, during reward anticipation the HA group had lower differentiation between the cues indicating potential to win or lose money. In contrast, the HA group showed a trend towards enhanced differentiation between incentive and neutral cues within the general motivational system. The multivariate ML approach demonstrated here allowed valence and salience to be disentangled. Furthermore, by preserving variance among trials within a task it was possible to examine dynamic cognitive states of individual participants and the relationship between their brain and behaviour. These benefits have potential clinical relevance as they could support patient diagnosis and stratification, where subtle individual differences in neural processing may be informative of a predisposing vulnerability to a disease and potential response to different treatment types.

## AUTHOR CONTRIBUTIONS

Conceptualisation and methodology: R.W., E.F. Data curation and investigation: M.K., R.W., Z.C. Formal analysis: M.K., E.F. Project administration: Z.C., M.K. Resources: C.R., E.F., A.M., R.W. Software: E.F. Supervision: E.F., C.R., A.M. Writing – original draft and visualisation: E. F, M.K., R.W. Writing – review and editing: C.R., G.G., A.M., E.F., R.W., Z.C. All authors critically reviewed content and approved the final version for publication.

## Supporting information




**Data S1.** Supporting Information

## Data Availability

The data that support the findings of this study are available on request from the corresponding author. The data are not publicly available due to privacy or ethical restrictions.
