# Supplementary material for: At‐risk alcohol users have disrupted valence discrimination during reward anticipation
Source: Addict Biol. 2022 Apr 7;27(3):e13174. doi: 10.1111/adb.13174 (PMC9286798; doi:10.1111/adb.13174)
Supplement: Supplementary file 1 — Data S1. Supporting Information [file ADB-27-0-s001.docx]

# Introduction

**Table 1. Testable Hypotheses for Cue-P3 and CNV time windows.** Details predefined outcomes for all pre-planned analyses which would support the testable hypotheses.

| **Hypotheses for valence sensitivity within Cue-P3 time window: 250 - 550ms after cue onset** | | | |
| --- | --- | --- | --- |
| **Hypothesis (H1)** | **Pre-defined outcome(s) which support H1** | **Pre-defined outcome(s) which do not support H1** | **Do our results give evidence for H1?** |
| Cue-P3 valence sensitivity in the LA group, such that gain amplitudes are enhanced compared with loss amplitudes. | Cue-P3 for gain cues is significantly larger than cue-P3 for loss cues. | No difference in cue-P3 amplitudes for the gain and loss cues.  OR  Cue-P3 for loss cues is significantly larger than cue-P3 for gain cues. | Yes – Section 3.2.1 |
| Lack of cue-P3 valence sensitivity in the HA group. | No difference in cue-P3 amplitudes for the gain and loss cues. | Cue-P3 for gain cues is significantly larger than cue-P3 for loss cues.  OR  Cue-P3 for loss cues is significantly larger than cue-P3 for gain cues. | Yes – Section 3.2.1 |
| Reduced cue-P3 valence sensitivity in the HA group compared with the LA group. | The 3 (condition) x 2 (group) mixed ANOVA reveals a significant group x condition interaction  Follow on tests reveal a larger difference in amplitude between the gain and loss condition for the LA group compared to the HA group | The 3 (condition) x 2 (group) mixed ANOVA does not give a significant group x condition interaction | No – Section 3.2.1 |
| Machine learning valence sensitivity in the LA group within the cue-P3 time window. | The multivariate machine learning valence (gains vs loss cues) discriminator performance (Az value) is significant within the cue-P3 time window. | The multivariate valence (gains vs loss cues) discriminator performance (Az value) is not significant within the cue-P3 time window. | Yes – Section 3.3.1 |
| Lack of machine learning valence sensitivity in the HA group within the cue-P3 time window. | The multivariate machine learning valence (gains vs loss cues) discriminator performance (Az value) is not significant within the cue-P3 time window. | The multivariate machine learning valence (gains vs loss cues) discriminator performance (Az value) is significant within the cue-P3 time window. | Yes – Section 3.3.1 |
| Reduced performance of valence ML discriminator in HA group compared with the LA group, within the cue-P3 time window. | ***Within the cue-P3 time window all three outcomes needed:***  The multivariate machine learning valence (gains vs loss cues) discriminator performance (Az value) for the ***LA group is significant***.  **AND**  The multivariate machine learning valence (gain vs loss cues) discriminator performance (Az value) for the ***HA group*** i***s not significant***.  **AND**   SPM1d analysis confirms there is a significant difference in LA group and HA group AZ values. | ***Within the cue-P3 time window:***  The multivariate machine learning valence (gains vs loss cues) discriminator performance (Az value) for the ***LA group is not significant***.  **AND**  The multivariate machine learning valence (gain vs loss cues) discriminator performance (Az value) for the ***HA group*** i***s significant***.  **AND**  SPM1d analysis does not confirm there is a significant difference in LA group and HA group AZ values. | Yes – Section 3.3.1 |
| **Hypotheses for salience sensitivity within CNV time window:  200ms before target onset** | | | |
| **Hypothesis (H1)** | **Pre-defined outcome(s) which support H1** | **Pre-defined outcome(s) which do not support H1** | **Do our results give evidence for H1?** |
| CNV salience sensitivity in the HA group, such that incentive amplitudes (gain & loss) are enhanced compared with neutral amplitudes. | CNV for incentive cues is significantly larger than CNV for neutral cues. | No difference in CNV amplitudes for the incentive and neutral cues.  OR  CNV for neutral cues is significantly larger than CNV for incentive cues. | Yes – Section 3.2.3 |
| Lack of CNV salience sensitivity in the LA group. | No difference in CNV amplitudes for the incentive and neutral cues. | CNV for incentive cues is significantly larger than CNV for neutral cues. | Yes – Section 3.2.3 |
| Increased CNV salience sensitivity in the HA group compared with the LA group. | The 3 (condition) x 2 (group) mixed ANOVA reveals a significant group x condition interaction  Follow on tests reveal a larger difference in amplitude between the incentive and neutral conditions for the HA group compared to the LA group | The 3 (condition) x 2 (group) mixed ANOVA does not give a significant group x condition interaction | No – Section 3.2.3 |
| Machine learning salience sensitivity in the HA group within the CNV time window. | The multivariate machine learning salience (incentive vs neutral cues) discriminator performance (Az value) is significant within the CNV time window. | The multivariate salience (incentive vs neutral cues) discriminator performance (Az value) is not significant within the CNV time window. | Yes – Section 3.3.3 |
| Lack of machine learning salience sensitivity in the LA group within the CNV time window. | The multivariate machine learning salience (incentive vs neutral cues) discriminator performance (Az value) is not significant within the CNV time window. | The multivariate machine learning salience (incentive vs neutral cues) discriminator performance (Az value) is significant within the CNV time window. | Yes – Section 3.3.3 |
| Reduced performance of salience ML discriminator in LA group compared with the HA group, within the CNV time window | ***Within the cue-P3 time window all three outcomes needed:***  The multivariate machine learning salience (incentive vs neutral cues) discriminator performance (Az value) for the ***HA group is significant***.  **AND**  The multivariate machine learning salience (incentive vs neutral cues) discriminator performance (Az value) for the ***LA group*** i***s not significant***.  **AND**   SPM1d analysis confirms there is a significant difference in HA group and LA group AZ values. | ***Within the cue-P3 time window:***  The multivariate machine learning salience (incentive vs neutral cues) discriminator performance (Az value) for the ***HA group is not significant***.  **AND**  The multivariate machine learning salience (incentive vs neutral cues) discriminator performance (Az value) for the ***LA group*** i***s significant***.  **AND**  SPM1d analysis does not confirm there is a significant difference in HA group and LA group AZ values. | No – Section 3.3.3 |

# Methods

## EEG pre-processing

The FASTER plug-in was first used to automatically identify and exclude non-neural independent components from the EEG data, and epochs containing large artifacts (e.g., muscle twitches). The ManualQC plug-in (<https://github.com/zh1peng/ManualQC>) was then used to visually inspect the remaining EEG to ensure good data quality and that any remaining noisy data were removed (e.g., eyeblinks, idiosyncratic muscle/movement, transient electrode artifacts and excessive alpha). We rejected artifacts using independent components analysis (ICA) (ICAs rejected: mean = 11.68, std. = 5.72); interpolated channels that had artifacts present throughout all epochs (channels interpolated: mean = 1.48, std.= 2.29); and removed specific epochs containing noise that was not present throughout all epochs (remaining epochs gain condition: mean = 40.29 trials, std. = 3.93; remaining epochs loss condition: mean =40.91 trials, std. = 4.45; remaining epochs neutral: mean = 40.54 trials, std. = 3.99).

## Event related potential analyses

For each anticipatory component with potential for valence sensitivity (i.e., P2, N2, Cue-P3a, Cue-P3b) we quantified ERP waveforms for the gain and loss conditions separately, over the electrode groups as defined in Section 2.5 of the main manuscript. For each component, a gain-minus-loss difference wave was then calculated as the voltage of each time point in loss condition’s ERP waveform subtracted from that of the gain condition’s ERP waveform. Finally, mean amplitudes for gain-minus-loss difference waves were calculated for each component across the time windows of interest.

To evaluate group differences, we conducted unpaired t-tests on mean amplitudes for the gain-minus-loss difference waves. We employed the Shapiro-Wilk test to evaluate whether the data were normally distributed within each group and used F-tests to evaluate equality of variances between the two groups. All ERP statistical analyses were carried out using the R software (version 4.0.5).

## Between group comparison of machine learning results

A scalar trajectory output statistic, SPM{t}, was calculated for each individual time node in the 1D continuum. In this case, a node represented the onset/offset of discrimination windows that occurred within the hypothesised time windows for valence and salience sensitivity. The calculation of SPM{t} shows only the magnitude of LA-Az to HA-Az differences, therefore it is not possible to accept or reject our null hypothesis (i.e., no group differences in Az) with this variable alone. We used RFT to calculate a critical threshold at which no more than 5% (α < 0.05) of equally smoothed random data would be expected to cross. The null hypothesis is rejected if the SPM{t} scalar trajectory crosses this critical threshold at any time node (Pataky et al., 2017).

# Results

## P2

For the LA group, we conducted a one-way repeated measures ANOVA on mean P2 and found a significant main effect of condition (F (2, 42) = 3.62, *p* = 0.035, η_p_^2^ = 0.147) (Fig. 4e). For the HA group, we conducted a one-way repeated measures ANOVA on mean P2 and found a significant main effect of condition (F (2, 42) = 4.87, *p* = 0.012, η_p_^2^ = 0.188) (Fig. 4f). Tukey post-hoc test revealed that there were no statistically significant differences between conditions in either group.

The 3 (condition) x 2 (group) mixed ANOVA revealed no significant differences between the groups for the P2 amplitude (F (1, 42) = 0.003, *p* = 0.955, η_p_^2^ < 0.001). There was however a significant main effect of condition on P2 amplitude (F (2, 84) = 8.26, *p* < 0.001, η_p_^2^ = 0.164). Tukey post-hoc tests showed significantly larger P2 amplitudes for the gain condition compared to the loss condition (p = 0.003) and the neutral condition compared to the loss condition (*p* = 0.040).

## N2

For the LA group, we conducted a one-way repeated measures ANOVA on mean N2, and the main effect of the condition did not reach significance (*F* (2, 42) = 0.57, *p* = 0.567, η_p_^2^ = 0.027) (Fig 4e). For the HA group, we conducted a one-way repeated measures ANOVA on mean N2 and found a significant main effect of condition (F (2, 42) = 5.73, *p* = 0.006, η_p_^2^ = 0.214) (Fig. 4f). Tukey post-hoc tests showed significantly larger N2 amplitudes for the gain condition compared to the loss condition (*p* = 0.025).

The 3 (condition) x 2 (group) mixed ANOVA revealed no significant differences between the groups for the N2 amplitude (F (1, 42) = 1.96, p = 0.169, η_p_^2^ = 0.045). There was however a significant main effect of condition on N2 amplitude (F (2, 84) = 4.12, p = 0.020, η_p_^2^ = 0.089). Tukey post-hoc tests showed significantly larger N2 amplitudes for the loss condition compared to the gain condition (*p* < 0.05).

## Gain-minus-Loss ERP difference waves

For all components, statistical tests confirmed the data were normally distributed within each group, and there was equality of variances between the two groups. Unpaired t-tests demonstrated there were no significant differences between the LA and the HA groups, for the gain-minus-loss difference waves within the time windows of the P2, N2, Cue-P3a and Cue-P3b components (Table 2) (Fig. 2a, 2b, 4e, 4f).

**Table 2. Results for gain-minus-loss difference waves**

| Component | t | df | *p*-value | Mean amplitude LA | Mean amplitude HA |
| --- | --- | --- | --- | --- | --- |
| P2 | 0.069 | 42 | 0.945 | 0.574 | 0.597 |
| N2 | 1.625 | 42 | 0.112 | 0.143 | 0.741 |
| Cue-P3a | -1.003 | 42 | 0.321 | 0.518 | 0.263 |
| Cue-P3b | -0.955 | 42 | 0.345 | 0.368 | 0.038 |

# Discussion

## Exploratory ERP analyses

Here, we conducted exploratory ERP analyses on the P2 and N2 to investigate whether these components were sensitive to between group differences in valence or salience processing. When combining data from both groups, the P2 was modulated by the task, such that amplitudes for gain and neutral conditions were enhanced compared with the loss condition. The P2 components for both LA group and the HA group were similarly modulated by the task (Fig. 4e-f) and consequently no between group differences were found.

Within the LA group, the N2 was insensitive to task manipulation (Fig. 4e), whereas within the HA group, the N2 showed valence sensitivity (i.e., amplitudes for loss > gain condition) (Fig. 4f). The N2 revealed potential for differentiating between the groups, with a hyperactive signal in the hazardous drinkers compared to controls, however, the mixed model ANOVA was not statistically significant between groups. This result may be the result of low power in the present study. It is therefore recommended that the N2 be included as a component of interest in follow on research exploring differences between alcohol dependent/at-risk drinkers and control subjects.
